# *Panax quinquefolius* saponins combined with dual antiplatelet drug therapy alleviate gastric mucosal injury and thrombogenesis through the COX/PG pathway in a rat model of acute myocardial infarction

**DOI:** 10.1371/journal.pone.0194082

**Published:** 2018-03-27

**Authors:** Na Kou, Mei Xue, Lin Yang, Ming-Xuan Zang, Hua Qu, Ming-Ming Wang, Yu Miao, Bin Yang, Da-Zhuo Shi

**Affiliations:** 1 Laboratory of Cardiology, Center of Cardiology, Xiyuan Hospital, China Academy of Chinese Medical Sciences, Beijing, China; 2 Xiyuan Hospital, Clinical College, Graduate school, Beijing University of Chinese Medicine, Beijing, China; Nagoya University, JAPAN

## Abstract

**Objectives:**

Previous studies have found that *Panax quinquefolius* saponins (PQS) combined with dual antiplatelet therapy (DAPT) of aspirin and clopidogrel enhances antithrombotic effects while reducing gastric mucosal injury induced by DAPT. We investigated the effects of the combined drug therapy (PQS+DAPT) through the COX/PG pathways.

**Methods:**

Acute myocardial infarction (AMI) was induced in Wistar rats by ligation of the left anterior descending (LAD) coronary artery, and the animals were randomly divided into Model, DAPT, and PQS+DAPT groups. Rats in the sham group did not undergo artery ligation. They were intragastrically treated for 14 days. Myocardial infarct size; myocardial pathology; platelet aggregation rate, CD62p activation, concentrations of thromboxane B_2_ (TXB_2_), 6-keto-PGF1α, tissue plasminogen activator (t-PA), and plasminogen activator inhibitor (PAI), the TXB_2_/6-keto-PGF1α ratio were measured. The ultrastructure of the gastric mucosa was observed by scanning electron microscopy. The expression of PGE_2_ and 6-keto-PGF1α in gastric mucosa was measured by radioimmunoassay, and levels of COX-1, COX-2, and VEGF in gastric mucosa were assessed using immunohistochemistry.

**Results:**

The addition of Panax quinquefolius saponins (PQS+DAPT) to standard DAPT therapy significantly decreased the myocardial infarct area, degree of myocardial lesions, TXB_2_ and PAI levels, and the TXB_2_/6-keto-PGF1α ratio, while increasing 6-keto-PGF1α and t-PA levels and reducing the degree of gastric mucosal injury. Expression of PGE_2_, 6-keto-PGF1α, COX-2, and VEGF in the gastric mucosa was upregulated in the PQS+DAPT group compared with the standard DAPT group.

**Conclusion:**

PQS increases the degree of DAPT inhibition of myocardial necrosis and antiplatelet effects in AMI rats, as well as reducing damage to the gastric mucosa caused by DAPT. The mechanism may be related to inhibition of TXB_2_ and PAI activity and elevation of 6-keto-PGF1α and t-PA levels in blood, and may be associated with upregulated expression of COX-2, PGE_2_, PGI_2_, and VEGF in gastric tissue.

## Introduction

Dual antiplatelet therapy (DAPT) consisting of aspirin and clopidogrel is the standard regimen for acute coronary syndrome (ACS) patients and patients who have undergone percutaneous coronary intervention (PCI) [1]. The relative risk of cardiovascular death, nonfatal myocardial infarction, and stroke is 20% lower with DAPT than with aspirin-only treatment [2]. However, DAPT results in a two- to three-fold higher incidence of gastrointestinal hemorrhage, compared with treatment with a single antiplatelet drug [3]. Therefore, side effects from drug therapy are therefore important factors related to poor prognosis of coronary heart disease (CHD) [4]. Achieving effective antiplatelet action while avoiding gastrointestinal damage or hemorrhage has become a focal point in selecting among treatments for patients with cardiovascular thrombotic disease.

Aspirin plays an important role in antiplatelet therapy as a cyclooxygenase (COX) inhibitor, and can irreversibly inhibit platelet COX-1 and reduce the synthesis of thromboxane A_2_ (TXA_2_) [5]. Aspirin is a non-steroidal drug, and can directly injure gastric mucosa and inhibit the activities of COX-1 and COX-2, leading to a reduction in prostaglandin (PG) and subsequently influence the regulation of gastrointestinal blood flow and mucous functions and weakening the gastric mucus bicarbonate barrier [6]. Clopidogrel, an adenosine diphosphate receptor antagonist, does not directly damage the digestive tract mucosa, but can inhibit platelet-derived growth factor (PDGF) and platelets from releasing vascular endothelial growth factor (VEGF), which blocks the neovascularization and subsequent repair of gastrointestinal mucosa [7].

**Fig 1:**
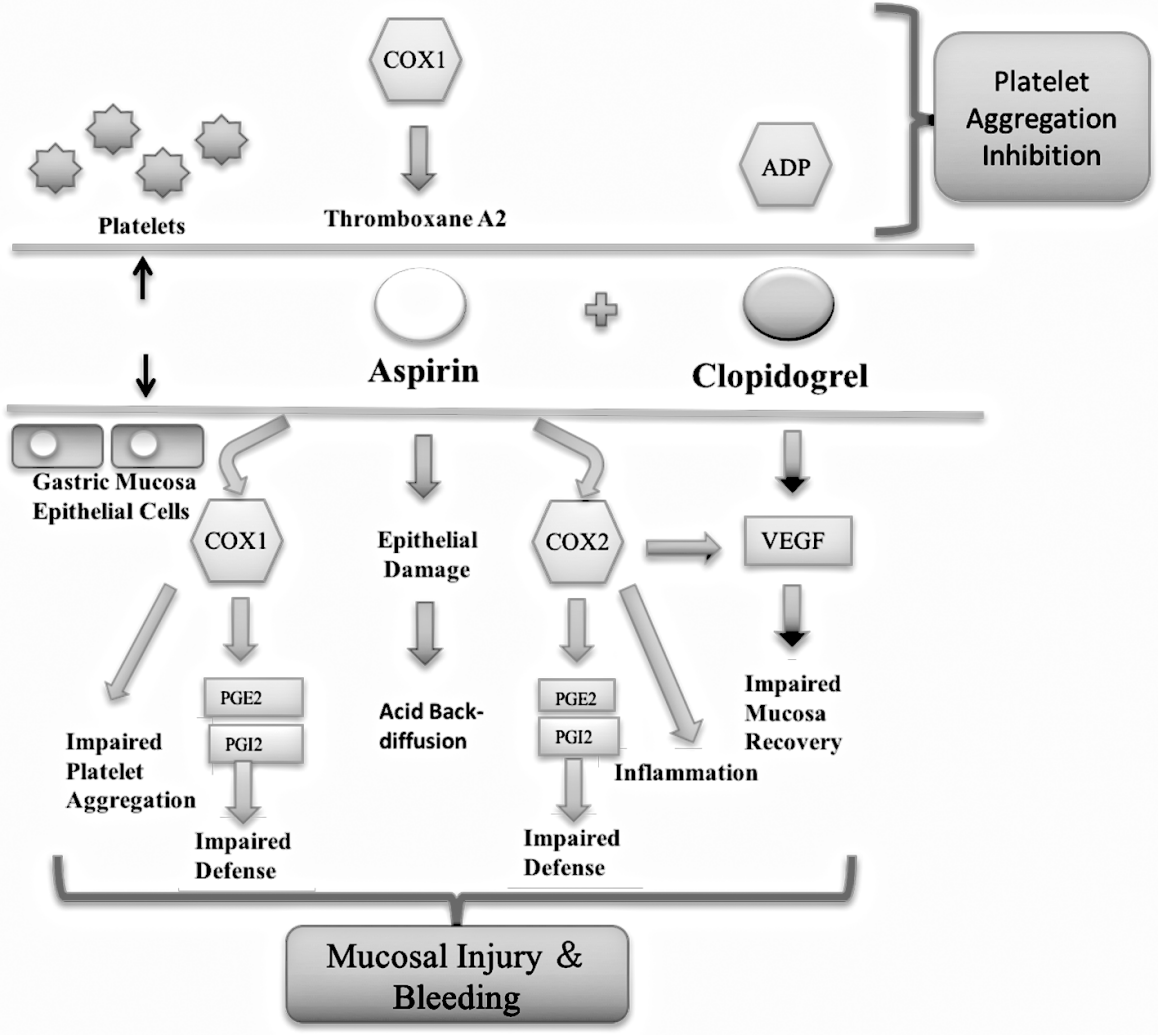
Mechanism of action of aspirin and clopidogrel on platelet aggregation and gastric mucosa injury.

Xinyue capsules (*Panax quinquefolium* saponins) contain stem and leaf extracts of American ginseng, and especially the saponins, including G-Rd, G-Rb_2_, G-Rb_3_, and P-F_1_. Xinyue capsules have been used to treat coronary heart disease for the past 20 years, and may effectively reduce the risk of angina and alleviate the patient’s clinical symptoms [8–9]. Our previous study confirmed that Panax quinquefolius Saponins (PQS) in combination with DAPT (PQS+DAPT) improved ventricular reconstruction in rats with acute myocardial infarction (AMI) rats and reduced gastric mucosa injury compared with DAPT alone [10]. Further research is required to explore the role of PQS+DAPT therapy in platelet activation and thrombosis of AMI rats, and determine whether PQS+DAPT therapy plays a protective role against gastric mucosa injury via COX-1-, COX-2-, PGI_2_-, PGE_2_-, or VEGF-related pathways. Therefore, we established an AMI models in rats, and observed the effects of PQS+DAPT therapy on platelet activation and mitigation of gastric mucosa injury to identify the mechanisms involved (Fig 1).

## Materials and methods

### Chemicals and reagents

Xinyue capsules (50 mg PQS/capsule) were provided by JiLin YiSheng Pharmaceutical Co. Ltd. (Jilin, China; SFDA approval certificate number: Z20030073). Enteric-coated aspirin tablets (100 mg) were obtained from Bayer Co. (Leverkusen, Germany; SFDA approval certificate number: J20080078). Clopidogrel tablets were obtained from Sanofi (Paris, France; SFDA approval certificate number: J20080090). Penicillin sodium injection (80 million IU/bottle) was purchased from North China Pharmaceutical Co. Ltd. (Hebei, China; SFDA approval certificate number: X1105313).

Triphenyltetrazolium chloride (TTC) was obtained from Sigma-Aldrich (St. Louis, MO, USA). Adenosine diphosphate (ADP) was obtained from Chrono-Log (Havertown, PA, USA). Fluorescent antibody PE-CD62p and FITC-CD61 were purchased from Bio-Rad Laboratories (Hercules, CA, USA). Thromboxane B_2_ (TXB_2_), 6-ketone prostaglandin F1α (6-keto-PGF1α), prostaglandin E_2_ (PGE_2_), tissue plasminogen activator (t-PA), and plasminogen activator inhibitor (PAI) radioimmunoassay kits were obtained from Huaying Engineering of Medicine and Biology Co. Ltd. (Beijing, China). Antibodies against COX-1 and COX-2 were obtained from Santa Cruz Biotechnology (Dallas, TX, USA). Antibodies against VEGF were obtained from Abcam (Cambridge, UK). Pentobarbital was obtained from Sinopharm Chemical Reagents (Beijing, China).

### Animals and treatments

Male Wistar rats (of weight approximately 180–200 g) were purchased from Beijing University Laboratory Animal Center (Beijing, China; certificate number SCXK [Jing] 2011–0004). The rats were allowed to acclimate for 7 d under standard animal care conditions with a temperature of 22–25°C and humidity of 55–65%. The animals had free access to a standard diet and tap water.

Rats were subjected to AMI by ligation of the left anterior descending (LAD) coronary artery as described previously [11], and were randomly assigned to sham, model, DAPT, and PQS+DAPT groups (n = 15 per group). The rats in the sham group did not undergo the LAD coronary artery ligation. Rats were administered 2 mL/kg/d distilled water (sham, model groups); 9 mg/kg/d aspirin and 6.25 mg/kg/d clopidogrel (DAPT group); 162 mg/kg/d Xinyue capsules, 9 mg/kg/d aspirin, and 6.25 mg/kg/d clopidogrel (PQS+DAPT group). All drugs were dissolved before use. DAPT and saline were administered orally to rats once daily at 9 AM, and PQS was administered orally to rats twice daily at 9:30 AM and at 7:00 PM for 14 d. Weight of rats was recorded before administered every morning. The Animal Care and Use Committee of Xiyuan Hospital of the China Academy of Chinese Medical Sciences approved the experimental protocol (Beijing, China; certificate number SYXK [Jing] 2015–0011).

The large number of indicators to be measured required that rats be anesthetized, and then euthanasized by blood collection. Rats were anesthetized with 1% pentobarbital 1 h after the last administered medication. Staining was carried out on 10 rats selected at random from each group, western blotting was carried out on six of the 15 rats from each group, and blood index detection was carried out on 12 rats selected at random from each group.

### TTC and hematoxylin and eosin staining

Based on reported methods [12], a stock solution of TTC reagent was prepared in 1% phosphate-buffered saline (PBS) at 25–30°C in the dark. Rat hearts were frozen at −20°C for 20 min, and transected into 5 slices approximately 2-mm thick perpendicular to the long axis. The sections were incubated in prepared TTC solution for 30 min at 37°C in the dark, and fixed with 4% paraformaldehyde for 5 min. The normal myocardium was red while the infarction area was white. The area of each region was calculated with Image-Pro Plus 6.0 (IPP 6.0; Roper Industries, Sarasota, FL, USA) on the scanned image, and the data are presented as the proportion of infarction area to the whole area of the heart.

Cardiac tissue specimens were placed in prepared 10% formaldehyde fixative; after dehydration, paraffin embedding, sectioning, coating, and dewaxing, the tissues were stained with hematoxylin and eosin, dehydrated, and sealed. Myocardium structure was observed under a microscope.

### Measurement of platelet aggregation

Blood was collected from the abdominal aorta and anticoagulated with sodium citrate (3.8%; 1 part anticoagulant to 9 parts blood). Platelet-rich plasma (PRP) was prepared by centrifuging the blood at 1,000 rpm for 8 min; blood was centrifuged at 3,000 rpm for 15 min to prepare platelet-poor plasma (PPP). The platelet concentration was adjusted to 1.8×10^9^/mL for PPP. According to the Born method [13], 0.3 mL PRP was placed in a cuvette and stirred with a rotor at 37°C for 5 min, after which 6 μL of ADP was added. Aggregation was measured with a platelet aggregometer (LBY-NJ4, Pulisheng Instrument Co. Ltd., Shanghai, China). Results were recorded as turbidimetry at maximal aggregation after the addition of an aggregating agent. Data are expressed as percentage of maximal aggregation.

### Measurement of CD62p expression

Two milliliters of abdominal aorta blood were taken and added to 3.8% sodium citrate for anticoagulation, shaken, and left to settle for 20 min. Five-microliter aliquots were then added to 5 μL of CD61FITC and 5 μL of CD62p PE, and incubated at 25–30°C for 20 min. PBS was added for a final volume of 1 mL. The solution was analyzed with a flow cytometer (NAVIOS, Beckman Coulter, Brea, CA, USA). Five microliters of PE-labeled immunoglobulin (IgG, CD62p negative) served as the control.

### Measurement of TXB_2_ and 6-keto-PGFIα levels in plasma and t-PA and PAI levels in serum

Concentrations of TXB_2_ (nonenzymatic metabolite of TXA_2_) and 6-keto-PGF1α (nonenzymatic metabolite of PGI_2_) in plasma, and t-PA and PAI in serum were measured using a radioimmunoassay (RIA). Abdominal aorta blood was drawn and centrifuged at 3,500 rpm for 10 min. Plasma and serum were collected and measured by RIA on an automatic RIA counter (XH-6020, Xi'an Nuclear Instrument Factory, Xi’an, China) according to the manufacturer’s instructions. The data are presented as pg/mL.

### Observation of gastric mucosa ultrastructure

Rat gastric antrum, 0.5 cm × 0.5 cm in size, was rinsed in saline and then rapidly removed to 2.5% glutaraldehyde (PBS preparation) for fixation. Tissue samples were placed in 1% osmic acid, and then underwent gradual dehydration with alcohol, isoamyl acetate replacement, critical-point drying, and metal spraying. A scanning electron microscope (S-520, Hitachi Ltd., Tokyo, Japan) was used to observe gastric mucosa ultrastructure. Observed the gastric mucosa ultrastructure changes by pathologists, on the principle of a double blindness.

### Measurement of PGE_2_ and 6-keto-PGFIα in gastric mucosa

The concentrations of PGE_2_ and 6-keto-PGF1α in gastric tissue were measured by RIA. The data are presented as pg/mL.

### Measurement of COX-1, COX-2, and VEGF in gastric mucosa

Rat gastric antrum tissue was extracted and prepared in paraffin sections. The paraffin sections were baked, deparaffinized, and hydrated in an ethanol gradient. This was followed by antigen retrieval and incubation in 3% H_2_O_2_ at 25–30°C. Primary antibodies were added for COX-1 (1:100), COX-2 (1:25), and VEGF (1:100). The samples were placed in a humidified box for incubation overnight at 4°C. Secondary antibodies were added dropwise the next day, followed by DAB color development, counterstaining, differentiation, dehydration, and section sealing. PBS served as the negative control for all antibodies, and a known positive tissue section served as a positive control. The sections were observed under an optical microscope, and five areas (four corners and one central area) were selected for imaging by a Motic MED 6.0 digital medical image analysis system (Beijing Mike Audi Image Technology Co. Ltd., Beijing, China) under 200× magnification. All images were analyzed by IPP 6.0. The expression of COX-1, COX-2, and VEGF was quantitatively analyzed with integrated optical density (IOD) as a testing index.

For western blotting, equal amounts of protein were loaded onto SDS-PAGE gels, and proteins were then transferred to nitrocellulose membranes (Millipore, Billerica, MA, USA). The membranes were blocked and incubated overnight at 4°C with the following antibodies: COX-1 (1:1,000), COX-2 (1:500), and GAPDH (1:500). The membranes were then incubated with secondary antibodies as follows: COX-1 (1:2,000), COX-2 (1:100), and GAPDH (1:500), for 1 h. Finally, the membranes were exposed and scanned, and the optical density of the bands (measured in arbitrary densitometry units) was determined using IPP 6.0. Results are expressed as a relative density ratio, normalized to the mean value of the Sham group. Two pathologists completed test using blind principle after each sample numbered.

### Statistical analysis

SPSS 17.0 was used for analysis (IBM SPSS, Armonk, NY, USA). All data are presented as mean ± standard deviation. ANOVA was applied following an F-test. Fisher’s least significant difference (LSD) method was used in cases of homogeneity of variance, while Welch’s correction was used in cases of heterogeneity of variance. *p*<0.05 was considered to indicate statistically significant differences.

## Results

### Survival status and weight changes in rats

Two rats of model group and one rat of DAPT group died within 14 days. At the onset of the experiment, the weight of rats in each group were not significantly different (*p*>0.05). However, weight in all three groups that had received arterial ligation rats significantly declined, relative to the Sham group after 14 days (*p*<0.05). There was no difference in weight among these three groups after 14 days (*p*>0.05, S1 Fig).

### PQS+DAPT reduced myocardium pathological changes and infarct size

**Fig 2:**
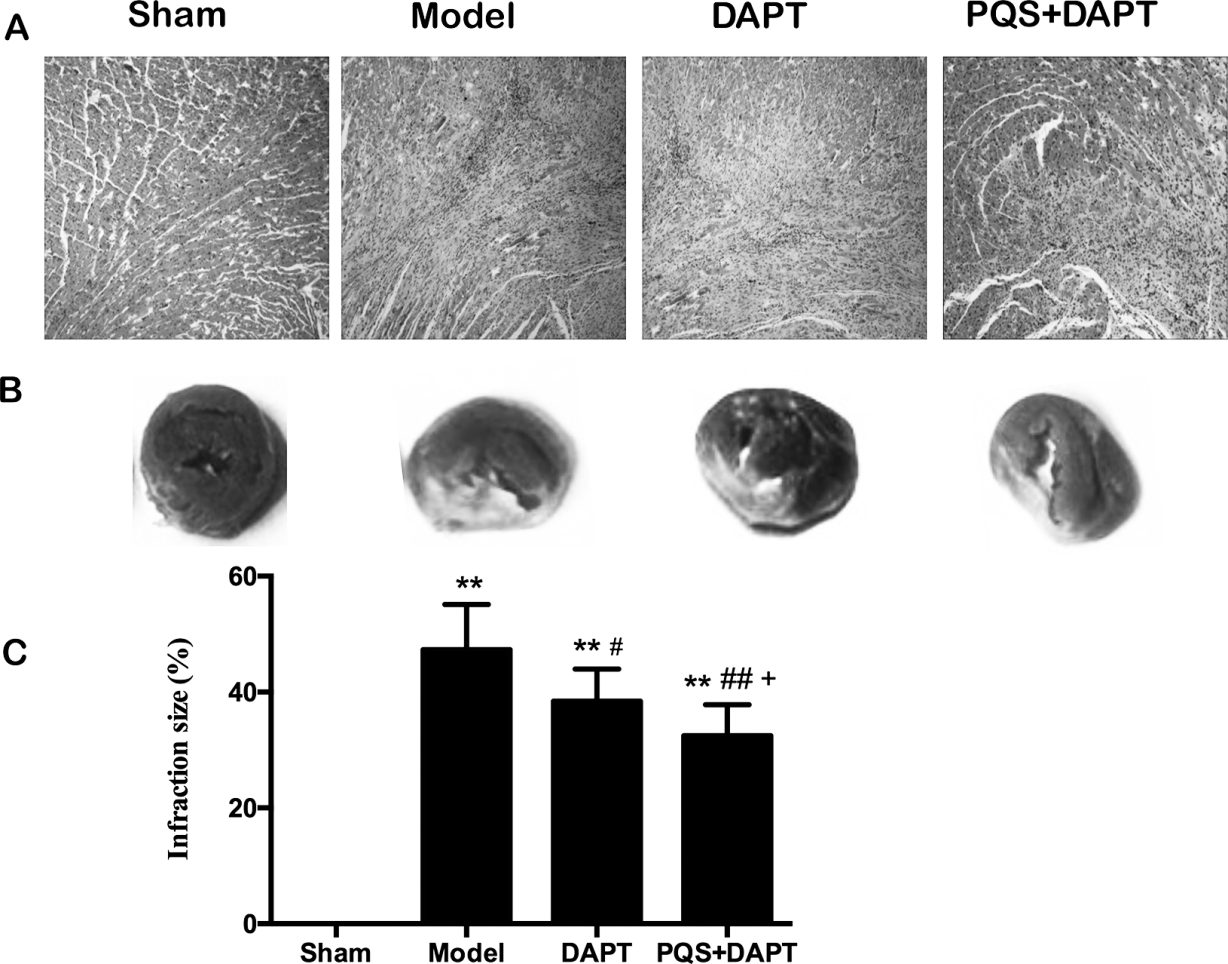
A: Myocardial pathological change in rats (hematoxylin and eosin, 10×). B, C: Comparison of myocardial infarct size using TTC staining, mean ± SD. N = 10. **p<0.01 vs. Sham. #*p*<0.05, ##*p*<0.01 vs. Model. +*p*<0.05 vs. DAPT.

Hematoxylin and eosin staining showed that the sham group displayed a centered cardiac myocyte nucleus, clear structure, no lipofuscin pigmentation in the nucleus, an orderly arrangement, clear transverse markings in the cardiac muscle fibers, no hyperplasia, no hypertrophy, no fibrosis, and no signs of edema or inflammatory cell infiltration in the mesenchyme. The model group displayed swelling, necrosis and karyolysis, disorderly arrangement in myocardial cells, breakage of cardiac muscle fibers, partial disappearance of myocardial cells, proliferation of some fibrous tissues, partial formation of map-like scar tissue, a distinct inflammatory reaction, and neutrophil infiltration. Pathological changes in the model group were similar to those in the DAPT group and PQS+DAPT group, but the lesion range was distinctly reduced in both treated groups. Interestingly, pathological changes in the PQS+DAPT group were smaller than in the DAPT group (Fig 2A).

TTC staining showed that the myocardial infarction area all three groups that had received arterial ligation increased significantly compared to the sham group (*p*<0.01). Compared with the model group, the infarction area of the DAPT group and the PQS+DAPT group was reduced by the therapy (*p*<0.01 and *p*<0.05); the infarction area of the PQS+DAPT group was further reduced compared with that of the DAPT group (*p*<0.05, Fig 2B and 2C).

### PQS+DAPT enhanced antiplatelet effects

**Fig 3:**
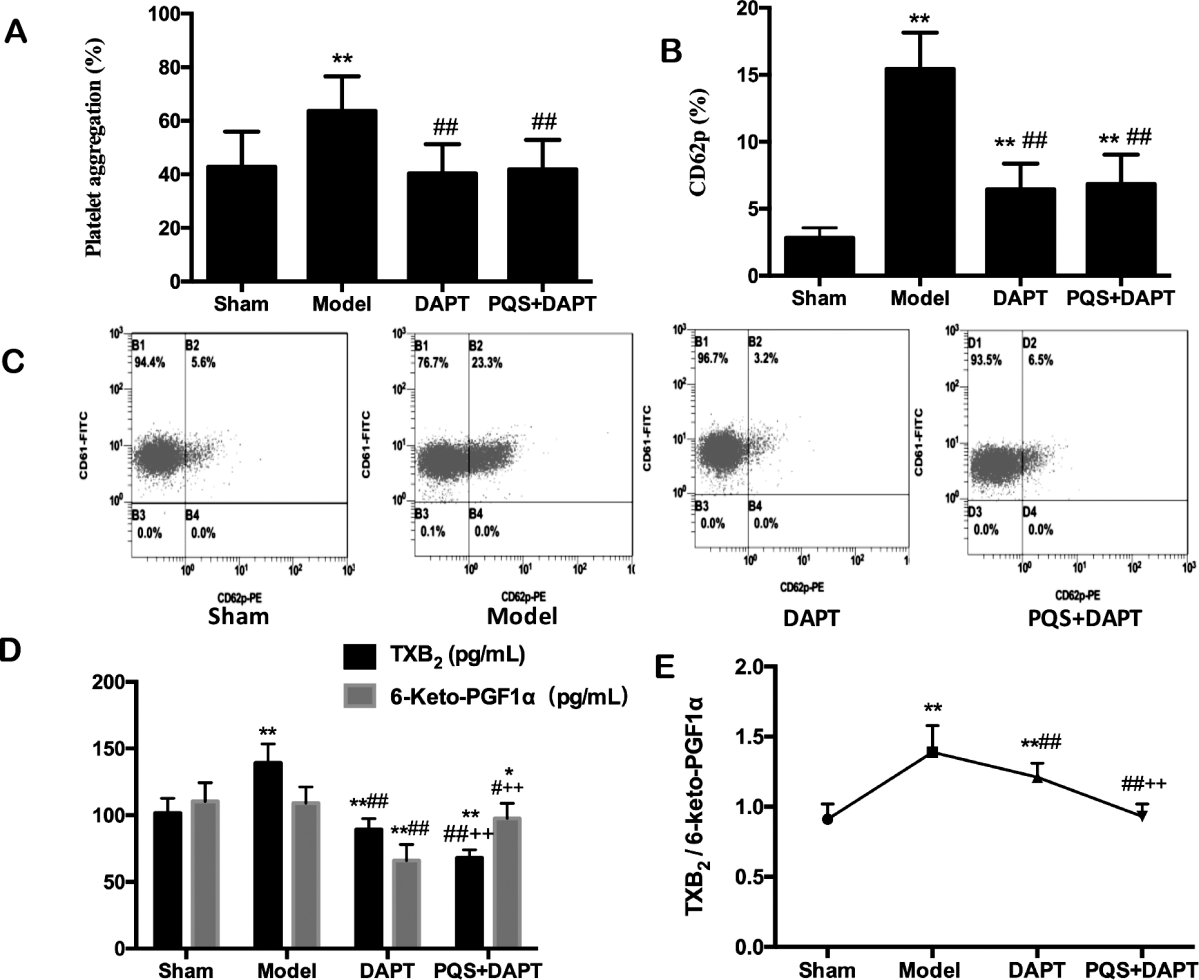
Effect of treatments on antiplatelets in rats. A: Platelet aggregation induced by adenosine diphosphate and measured by turbidimetry, mean ± SD. N = 12. **p<0.01 vs. Sham. ##*p*<0.01 vs. Model. B, C: CD62p measured by flow cytometry, mean ± SD. N = 12. **p<0.01 vs. Sham. ##*p*<0.01 vs. Model. D: TXB_2_ and 6-keto-PGF1α levels in rat plasma, measured using a radioimmunoassay, mean ± SD. N = 12. *p<0.05, **p<0.01 vs. Sham; #*p*<0.05, ##*p*<0.01 vs. Model; ++*p*<0.01 vs. DAPT. E: TXB_2_/6-keto-PGF1α ratio, mean ± SD. N = 12. **p<0.01 vs. Sham; ##*p*<0.01 vs. Model; ++*p*<0.01 vs. DAPT.

Compared with the sham group, the platelet aggregation rates increased significantly in the model group, with notable increases in CD62p expression, TXB_2_ concentrations, and the TXB_2_/6-keto-PGF1α ratio (*p*<0.01). 6-keto-PGF1α levels were not statistically significant (*p*>0.05). Compared with the model group, the platelet aggregation rates (*p*<0.01), the CD62p expression (*p*<0.01), the TXB_2_ levels (*p*<0.01), the 6-keto-PGF1α levels (*p*<0.01 and *p*<0.05), and the TXB_2_/6-keto-PGF1α ratio (*p*<0.01) were significantly lower in both the DAPT group and the PQS+DAPT groups. Platelet aggregation rates and CD62p expression in the PQS+DAPT group were not statistically different from those in the DAPT group (*p*>0.05, Fig 3A and 3B); TXB_2_ levels were significantly lower in the PQS+DAPT group (*p*<0.01), as were 6-keto-PGF1α concentrations and the TXB_2_/6-keto-PGF1α ratio (*p*<0.01, Fig 3C).

### PQS+DAPT increased t-PA and decreased PAI

**Fig 4:**
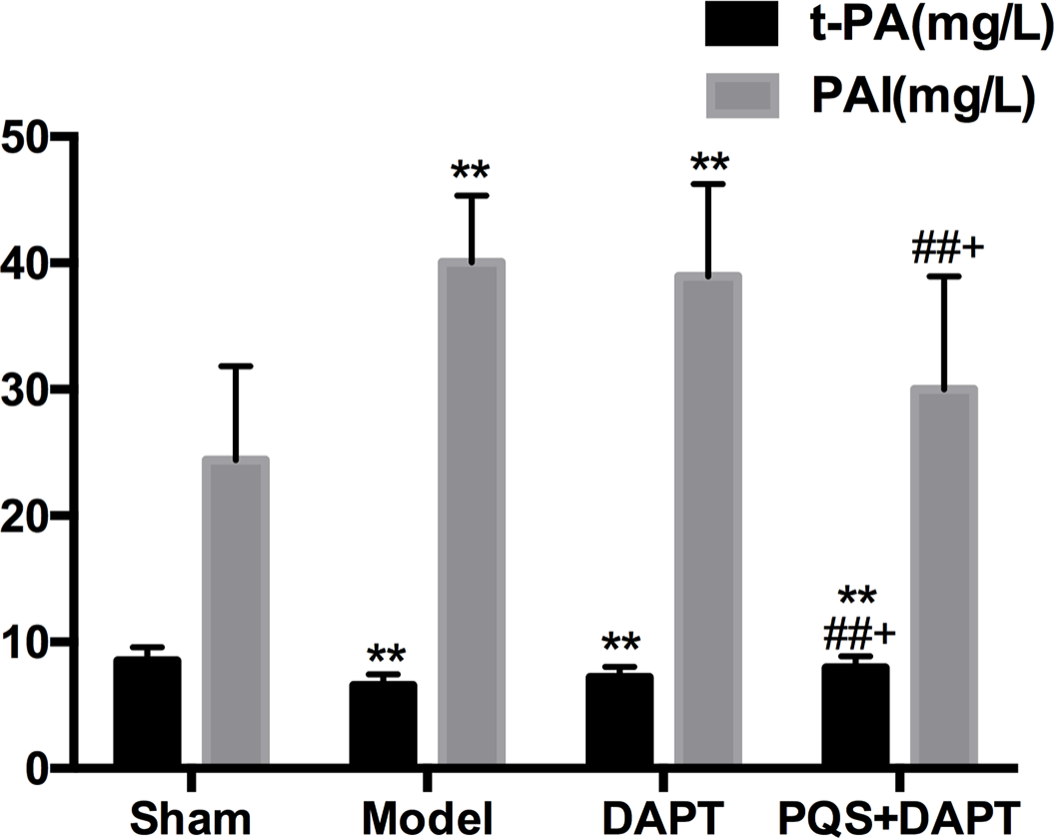
Effect of treatments on t-PA and PAI in rats. Blood was drawn from the abdominal aorta and anticoagulated with sodium citrate. Plasma samples were prepared by centrifugation and analyzed by radioimmunoassay, mean ± SD. N = 12. **p<0.01 vs. Sham. ##*p*<0.01 vs. Model. +*p*<0.05 vs. DAPT.

Compared with the sham group, t-PA levels in the model group decreased by 1.65 mg/L (*p*<0.01), while PAI levels increased by 15.62 mg/L (*p*<0.01). PAI and t-PA levels in the DAPT group were not statistically different from those in the model group (*p*>0.05), however, t-PA levels increased significantly (*p*<0.01) and PAI levels decreased significantly (*p*<0.01) in the PQS+DAPT group compared with the model group. Compared with the DAPT group, t-PA levels increased, and PAI levels decreased in the PQS+DAPT group (*p*<0.05, Fig 4).

### PQS reduced gastric mucosa injury induced by DAPT

**Fig 5:**
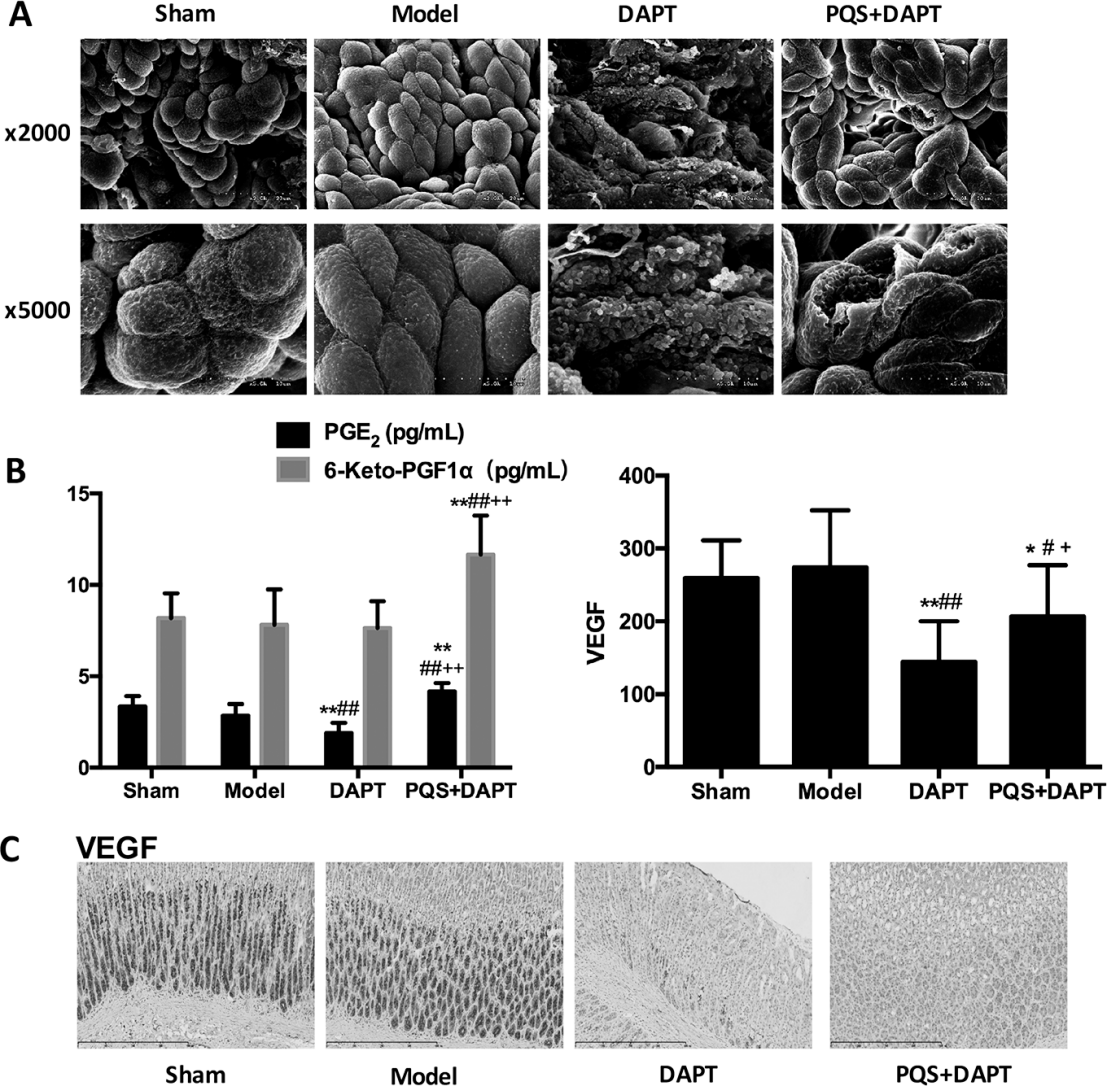
A: Gastric mucosa ultrastructure imaged by electron microscope, 2,000×, scale bar: 20 μm; 5,000×, scale bar:10 μm. B: Expression of PGE_2_ and 6-keto-PGFIα in rat gastric mucosa, mean ± SD. N = 12. **p<0.01 vs. Sham. ##*p*<0.01 vs. Model. ++*p*<0.01 vs. DAPT. Effect of VEGF levels in rat gastric mucosa, analyzed by immunohistochemistry. mean ± SD. N = 12. *p<0.05, **p<0.01 vs. Sham. #*p*<0.05, ##*p*<0.01 vs. Model, +*p*<0.05 vs. DAPT. C: VEGF expression in rat gastric mucosa, analyzed by immunohistochemistry. Scale bar: 400 μm.

In the sham group and model groups, mucosal epithelial cells were arranged well, similar in size and regular in shape, without visible damage. In the DAPT group, much of the basement membrane was damaged, cell size varied, cells displayed a fuzzy boundary, the surface plasma membrane was absent, the subsurface structure was completely exposed, and variable secretory granules were present. In the PQS+DAPT group, some of the damaged mucous cells were repaired, and only a small number of epithelial cells were ruptured (Fig 5A).

### PQS restored DAPT-inhibited expression of PGE_2_ and 6-keto-PGFIα in gastric mucosa

AMI rats in the model group did not display a statistically significant difference in the expression of PGE_2_ or 6-keto-PGF1α in the gastric mucosa compared with the sham group (*p*>0.05). Compared with the model group, the expression of PGE_2_ was reduced in the DAPT group (*p*<0.01), but 6-keto-PGF1α expression was not (*p*>0.05); the expression of PGE_2_ did not differ in the PQS+DAPT group. 6-keto-PGF1α expression increased (*p*<0.01) in the PQS+DAPT group, but not in the DAPT group (*p*>0.05). Compared with the DAPT group, PQS+DAPT treatment resulted in greatly upregulated expression of PGE_2_ and 6-keto-PGF1α in the gastric mucosa (*p*<0.01, Fig 5B).

### PQS restored DAPT-inhibited expression of VEGF, COX-1, and COX-2 in gastric mucosa

There was no significant difference between the sham group and the model group in the expression of VEGF (*p*>0.05). Compared with the model group, VEGF expression in gastric mucosa was notably downregulated in the DAPT group (*p*<0.05) and the PQS+DAPT group (*p*<0.01). Compared with the DAPT group, the expression of VEGF was notably upregulated in the PQS+DAPT group (*p*<0.05, Fig 5B and 5C).

**Fig 6:**
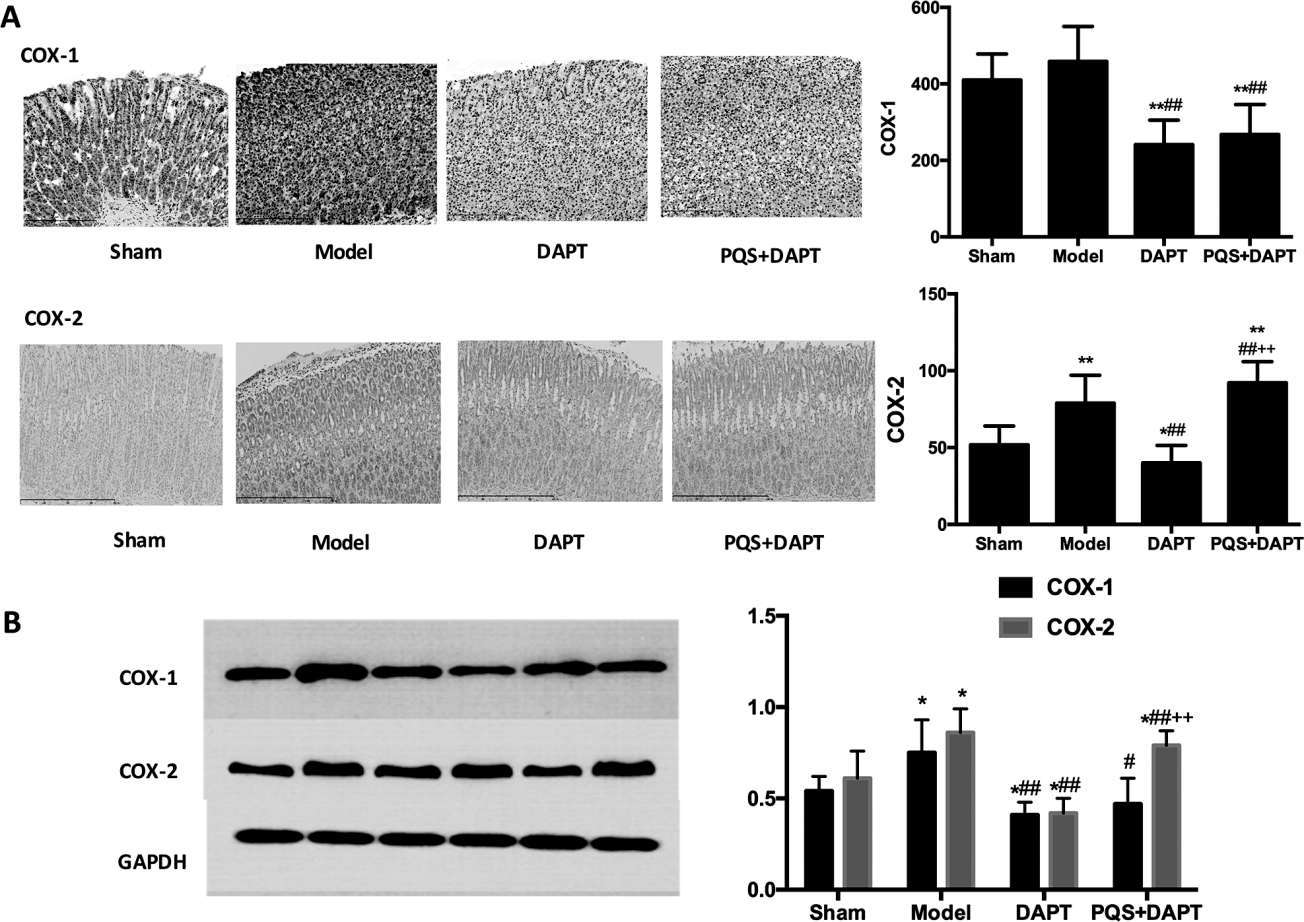
A: Effect of treatments on COX-1 and COX-2 levels in rat gastric mucosa, analyzed by immunohistochemistry. Scale bar: 400 μm, mean ± SD. N = 12. *p<0.05, **p<0.01 vs. Sham; ##*p*<0.01 vs. Model; ++*p*<0.01 vs. DAPT. B: Effect of treatments on the expression of COX-1 and COX-2 in rat gastric mucosa, analyzed by western blotting. Mean ± SD. N = 6. *p<0.05 vs. Sham; #*p*<0.05, ##*p*<0.01 vs. Model; ++*p*<0.01 vs. DAPT.

There was no significant difference between the sham group and the model group in the expression of COX-1 (*p*>0.05), but COX-2 expression increased significantly (*p<*0.05). Compared with the model group, the expression of COX-1 and COX-2 in gastric mucosa was significantly downregulated in the DAPT group and PQS+DAPT group (*p*<0.05 and *p*<0.01, respectively). Compared with the DAPT group, the expression of COX-2 was notably upregulated in the PQS+DAPT group (*p*<0.05), while the expression of COX-1 did not differ (*p*>0.05, Fig 6).

## Discussion

The present study found that DAPT therapy could reduce pathological damage from myocardial infarction in rats and effectively inhibit platelet activation, but also that DAPT damages gastric mucosa by inhibiting the expression of COX-1, COX-2, PGE_2_, PGI_2_, and VEGF. Notably, the present study also showed that PQS enhanced the protective and antithrombotic effects of DAPT therapy in myocardial infarction; the mechanism may be related to enhancing the inhibitory effects of aspirin on the COX-1/TXA_2_ pathway in platelets, the upregulation of the COX/PGI_2_ pathway, and the activation of the fibrinolytic system. PQS reduced lesions caused by DAPT therapy; this effect may be mainly attributable to the upregulation of COX2, PGI_2_, PGE_2_, and VEGF in gastric mucosa.

we studied the impact of a variety of treatments on the ventricular remodeling after myocardial infarction. The research indicates that the area of myocardial infarction for the PQS+DAPT group of AMI rat after 14 days is smaller than that of AMI group and proves that PQS can reduce the collagen content of cardiac muscle tissue after myocardial infarction and improve ventricular remodeling [14]. In addition, PQS can reduce the myocardial apoptosis rate in non-infarct areas [15]. The greatest myocardial necrosis was observed in the Model group after 14 d of treatment, and injury and necrosis and the myocardial infarction area were reduced in the DAPT group. Consistent with previous findings, the PQS+DAPT group exhibited less myocardial degeneration and necrosis than the DAPT group.

Arachidonic acid synthesizes TXA_2_ and PGI_2_ under the effects of COX [16]. TXB_2_ is a stable metabolite of TXA_2_ that can promote vasoconstriction and platelet aggregation [17]. In contrast, 6-keto-PGF1α is a stable metabolite of PGI_2_ that can inhibit vasoconstriction and platelet aggregation. The balance between these two metabolites plays an important part in maintaining blood circulation and microcirculation [18]. Aspirin irreversibly inhibits COX-1 activity in platelets and reduces TXA_2_ generation, thus inhibiting platelet aggregation and release [19]. Clopidogrel selectively inhibits ADP and its platelet receptors and the subsequent ADP-mediated activation of the GPIIb/IIIa complex; the combined application of two drugs with different mechanisms can enhance antiplatelet effects [20]. This study found an increased platelet aggregation rate, upregulated expression of CD62p, and elevated concentrations of TXB_2_ in rats with myocardial infarction. Decreased concentrations of 6-keto-PGF1α, an elevated TXB_2_/6-keto-PGF1α ratio, decreased serum t-PA levels, and higher PAI concentrations were also found after the 14-d treatment. This suggests that a variety of active substances were synthesized and released after platelet activation; the balance of TXA_2_ and PGI_2_ was disturbed, causing thrombosis and subsequent activation of the fibrinolytic system.

Our results suggest that DAPT therapy significantly inhibited platelet activation, while exerting no notable effects on the fibrinolytic system, and PQS enhances the antithrombotic effects of DAPT therapy by increasing aspirin inhibition of the COX-1/TXA_2_ pathway in platelets, improving the TXA_2_/PGI_2_ balance, and activating the fibrinolytic system.

In this study, PQS reduced the pathological injury to the gastric mucosa induced by DAPT. The expression of COX-1 and COX-2 in the gastric mucosa can catalyze arachidonic acid to produce PGE_2_, PGI_2_, and other protective factors [21–22]. PGE_2_ and PGI_2_ can inhibit the secretion of gastric acid, promote the secretion of gastric mucus and hydrocarbonate, dilate blood vessels, increase the blood flow volume in gastric mucosa, and promote the renewal and proliferation of epithelial cells in the gastric mucosa [6]. VEGF can stimulate the proliferation of epithelial cells, formation of blood capillaries, and generation of granulation tissue, taking part in the defense and repair of gastric mucosa [23]. Aspirin inhibits COX-1 and COX-2 activity in the gastric mucosa, leading to a decreased synthesis of PGE_2_ and PGI_2_ and other factors, undermining the defense function of the gastric mucosa barrier [24]. In the presence of gastric acid, fat-soluble aspirin cannot be ionized. Instead, it dissolves in the gastric juice, directly damaging the gastric mucosa barrier and penetrating the epithelial cell membrane [25]. Clopidogrel can directly reduce the synthesis of VEGF. The combination of aspirin and clopidogrel delays the repair of gastric mucosa [26].

The increased expression of COX-2 in gastric tissue of the model group that we observed may have been associated with damage to the gastric mucosa and stimulation of inflammatory reactions following AMI. The expression of COX-1, COX-2, PGE_2_, PGI_2_, and VEGF in the gastric mucosa in the DAPT group was downregulated. The expression of COX-2 was positively correlated with VEGF synthesis, and the inhibition of the COX/VEGF pathway may be an important mechanism of injury following the combined use of aspirin and clopidogrel. Compared with the DAPT group, the PQS+DAPT group exhibited an elevated expression of COX-2, PGE_2_, PGI_2_, and VEGF, while COX-1 expression was not significantly upregulated, indicating that PQS upregulates the expression of COX-2 instead of COX-1; this results in an increase in PGE_2_, PGI_2_, and VEGF expression, fortifying the barrier defense and repair function of the gastric mucosa.

## Conclusions

PQS enhances the inhibitory role of DAPT in protecting against myocardial necrosis and thrombosis in rats that have received AMI, possibly this may be attributable to the inhibition of the COX-1/TXA2 pathway in platelets, the upregulation of the COX/PGI_2_ pathway, and the activation of the fibrinolytic system by PQS. PQS alleviates DAPT-induced damage to the gastric mucosa, and the underlying mechanism may be associated with the upregulation of COX-2/PGE_2_, PGI_2_, and VEGF expression. Taken together, our findings indicate that PQS exerts a favorable effect on antiplatelet and gastric mucosa protection, which provides a new complementary approach to improving the prognosis of thrombotic diseases in combination with DAPT.

## Supporting information

S1 Fig Weight of rats on the 1st day and the 14th day after intragastric administration. N = 15, but 2 rats of model group died respectively on the 2nd day and the 4th day after intragastric administration, 1 rat of DAPT group died on the 3rd day after intragastric administration. Mean ± SD, *p<0.05 vs. Sham.
(TIF)
