# Supplementary figures and images for: Panax quinquefolius saponins combined with dual antiplatelet drug therapy alleviate gastric mucosal injury and thrombogenesis through the COX/PG pathway in a rat model of acute myocardial infarction
Source: PLoS One. 2018 Mar 27;13(3):e0194082. doi: 10.1371/journal.pone.0194082 (PMC5870954; doi:10.1371/journal.pone.0194082)

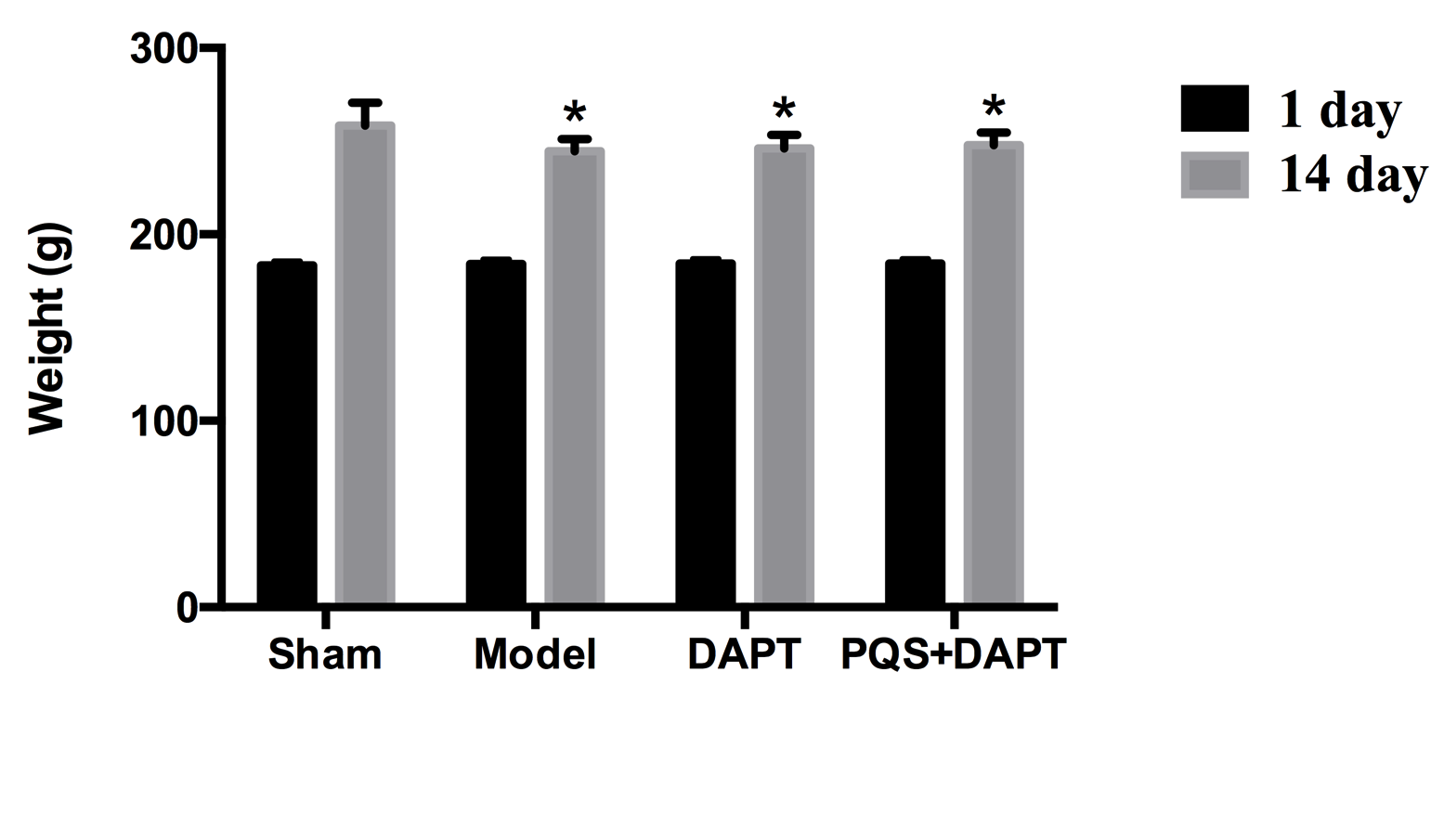

Supplement: S1 Fig — N = 15, but 2 rats of model group died respectively on the 2nd day and the 4th day after intragastric administration, 1 rat of DAPT group died on the 3rd day after intragastric administration. Mean ± SD, *p<0.05 vs. Sham. (TIF) [file pone.0194082.s001.tif]
